# Testing eukaryotic routes for heterologous production of ketocarotenoids in cyanobacteria

**DOI:** 10.1093/sumbio/qvaf008

**Published:** 2025-06-03

**Authors:** Anika V Mahdi, Dennis N Wittmann, Sayali S Hanamghar, Oskar Zorć, Jialan Cao, G Alexander Groß, Poul Erik Jensen, David A Russo, Julie A Z Zedler

**Affiliations:** Synthetic Biology of Photosynthetic Organisms, Matthias Schleiden Institute for Genetics, Bioinformatics and Molecular Botany, Friedrich Schiller University Jena, 07743 Jena, Germany; Synthetic Biology of Photosynthetic Organisms, Matthias Schleiden Institute for Genetics, Bioinformatics and Molecular Botany, Friedrich Schiller University Jena, 07743 Jena, Germany; Synthetic Biology of Photosynthetic Organisms, Matthias Schleiden Institute for Genetics, Bioinformatics and Molecular Botany, Friedrich Schiller University Jena, 07743 Jena, Germany; Synthetic Biology of Photosynthetic Organisms, Matthias Schleiden Institute for Genetics, Bioinformatics and Molecular Botany, Friedrich Schiller University Jena, 07743 Jena, Germany; Department of Physical Chemistry and Microreaction Technology, Institute of Chemistry and Bioengineering, Ilmenau University of Technology, 98693 Ilmenau, Germany; Department of Physical Chemistry and Microreaction Technology, Institute of Chemistry and Bioengineering, Ilmenau University of Technology, 98693 Ilmenau, Germany; Department of Food Science, University of Copenhagen, 1958 Frederiksberg, Denmark; Bioorganic Analytics, Institute for Inorganic and Analytical Chemistry, Friedrich Schiller University Jena, 07743 Jena, Germany; Synthetic Biology of Photosynthetic Organisms, Matthias Schleiden Institute for Genetics, Bioinformatics and Molecular Botany, Friedrich Schiller University Jena, 07743 Jena, Germany

**Keywords:** astaxanthin, β-carotene ketolase, astaxanthin synthase CrtS, canthaxanthin, cyanobacteria, ketocarotenoids

## Abstract

Pigments and dyes are widely used in multiple industries. Ketocarotenoids are a highly sought-after group of pigments with a dark-red hue and strong antioxidant properties. Currently, chemical synthesis is the major route for ketocarotenoid production. However, the environmental risks and large demand have led to a call for the development of more sustainable sources. Cyanobacteria have promising properties for sustainable ketocarotenoid production: Some strains show fast growth, are genetically tractable, and natively produce ketocarotenoid precursors. Despite a few promising studies, engineering ketocarotenoid production in cyanobacteria is so far underexplored. Here, we expand the biosynthetic routes for ketocarotenoids by testing the astaxanthin synthase CrtS of *Xanthophyllomyces dendrorhous* and the β-carotene ketolase CrBKT from *Chlamydomonas reinhardtii*. While CrtS was successfully produced in *Synechocystis* sp. PCC 6803, no changes in ketocarotenoid profiles were detected. On the other hand, expression of *CrBKT* in *Synechococcus elongatus* UTEX 2973 and *Synechocystis* sp. PCC 6803 led to the production of both canthaxanthin (0.12% and 0.1% of dry cell weight, respectively) and astaxanthin (0.012% and 0.004% of dry cell weight, respectively). Looking forward, expression of *CrBKT* is a promising route to produce ketocarotenoids in cyanobacteria. But further work is needed to increase strain stability and product yields.

**Sustainability Statement**

The recent increase in awareness regarding the health benefits of natural pigments (e.g. carotenoids) has led to an unprecedented demand. In this study, we explore different routes to produce ketocarotenoids, pigments with exceptional antioxidant properties, in green hosts such as cyanobacteria. Our findings show that the sustainable production of pigments, solely from CO_2_ and light, is a promising way to take on current global challenges addressed by the United Nations Sustainable Development Goals One Health (SDG3), Industry, Innovation, and Infrastructure (SDG9), and Climate Action (SDG13).

## Introduction

Colors are a fundamental aspect of human perception. Owing to this, pigments have become some of the most sought-after industrial products. Currently, the market is dominated by synthetic pigments. But due to environmental- and health-related concerns, there has been a major push in the research and development of natural pigment sources (Qin et al. 2023). Of all the natural pigments, carotenoids are among the most promising. Not only can they be used to produce natural shades of red and yellow, but their excellent antioxidant properties have applications in food, nutraceuticals, and pharmaceuticals. In upcoming years, the carotenoid global market is projected to reach the billion-dollar range (Ángeles et al. 2025).

Among the carotenoids, ketocarotenoids have a particularly high market demand. Ketocarotenoids are ketolated xanthophylls (i.e. oxygenated carotenoids with a keto moiety) with a red hue. The most popular ketocarotenoids are astaxanthin (3,3′-dihydroxy-β,β-carotene-4,4′-dione) and, to a lesser extent, canthaxanthin (β,β-carotene-4,4′-dione). In recent years, astaxanthin has become popular for its strong antioxidant properties, ten times more effective than β-carotene, zeaxanthin, or canthaxanthin and 100 times stronger than α-tocopherol (Schmidt et al. 2011). Because of these properties, astaxanthin is considered a high-value compound with applications in multiple industries, particularly in animal feed and aquaculture [e.g. as a feed additive at salmon farms to achieve natural coloration (Schmidt et al. 2011)]. To produce astaxanthin, there are several different approaches. Synthetically produced astaxanthin has a 1:2:1 ratio of three optical isomers: (3S, 3′S), (3R, 3′S), and (3R, 3′R). However, the mixture results in 20–50 times weaker antioxidant activity than natural astaxanthin, which typically exists either in (3S, 3′S) or (3R, 3′R) form (Fang et al. 2019).

In nature, astaxanthin is mainly found in the marine environment in microalgae and the shells of certain crustaceans. However, other natural sources exist. These include a few species of the land plant *Adonis*, carotenogenic bacteria (e.g. *Brevundimonas* spp., *Paracoccus* spp.), and certain yeasts (Zhang et al. 2020). The most important native astaxanthin producers are the yeast *Xanthophyllomyces dendrorhous* (the sexual state of *Phaffia rhodozyma*) and the microalga *Haematococcus pluvialis* (*H. lacustris*) (Fang et al. 2019, Zhang et al. 2020). Both *X. dendrorhous* and *H. pluvialis* can natively accumulate between 3% and 4% of dry weight of astaxanthin. However, *H. pluvialis* has the advantage of producing exclusively the (3S, 3′S) isomer, which exhibits the strongest antioxidant properties, solely from CO_2_, H_2_O, and nutrients (Shah et al. 2016). Despite the potential, astaxanthin production in this alga has major disadvantages: *H. pluvialis* grows slowly and requires a two-step astaxanthin induction process. First, the alga is cultured in a green vegetative phase until a high cell number is reached. Then, the formation of hematocysts (aplanospores) is induced by stress (e.g. nutrient deficiency, high light, pH, or salt stress). *H. pluvialis* spores then accumulate astaxanthin and can be harvested. The induction phase is costly and logistically complex when working at scale, and is the major reason for the lack of economic competitiveness (Shah et al. 2016). Therefore, current production systems with native astaxanthin-producing organisms do not meet the requirements of an efficient industrial production system and are unable to reach the high demand for natural astaxanthin. A promising route to overcome this challenge is the genetic engineering of model photoautotrophic hosts to generate highly efficient, sustainable astaxanthin-producing organisms.

**Figure 1.**
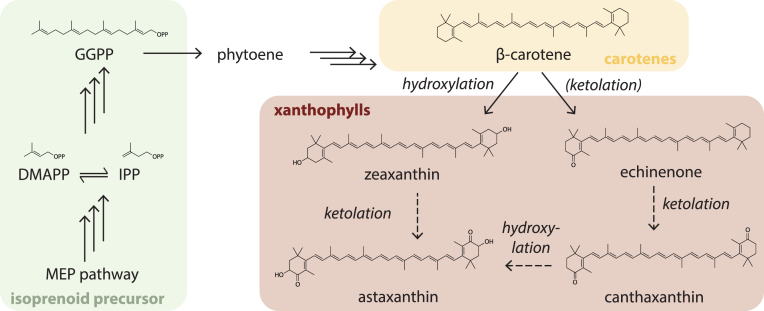
Overview of potential β-carotene-derived ketocarotenoid biosynthesis pathways by combination of endogenous pathways and introduction of heterologous genes in unicellular cyanobacteria. The carotenoid precursors are derived from the methylerythritol phosphate (MEP) pathway by condensation of dimethylallyl diphosphate (DMAPP) and isopentenyl diphosphate (IPP) units. The first step toward carotenoids is the formation of phytoene from two geranylgeranyl diphosphate (GGPP) molecules. This is followed by desaturase, isomerase, and terminal ring cyclization reactions to form carotenes, including β-carotene. Several different xanthophylls are then derived from sequential hydroxylation and ketolation reactions of β-carotene. Only main xanthophylls are shown and additional routes exist. Not all unicellular cyanobacteria are capable of natively producing echinenone (Liang et al. 2006), while filamentous cyanobacteria can also natively produce canthaxanthin (Sandmann 2021). Dashed lines show non-native routes.

To date, “green” heterologous astaxanthin production has been established in cyanobacteria (reviewed in Pagels et al. 2021), microalgae (reviewed in Goold et al. 2024), and various higher plants (reviewed in Rodriguez-Concepcion et al. 2018) using a variety of ketolases and hydroxylases. Microalgae and plants are still limited by relatively long genetic engineering cycles. In addition, upscaling is challenging due to low biomass accumulation and consequent low volumetric and areal productivities. Cyanobacteria, on the other hand, have taken a premier role in sustainable biotechnology. This is mainly due to their ease of engineering, fast growth, sustained biomass accumulation, and ability to produce a variety of industrially relevant small molecules and proteins (recently reviewed in Jodlbauer et al. 2021, Li et al. 2022, Melis et al. 2023, Toepel et al. 2023). To produce astaxanthin and other ketocarotenoids in cyanobacteria, the endogenous carotenoid biosynthesis pathway has to be extended. This can be divided into three major parts (Fig. 1): First, the native generation of the isoprenoid precursor via condensation of isopentenyl diphosphate and dimethylallyl pyrophosphate C_5_ building blocks for the formation of geranylgeranyl pyrophosphate (GGPP). Second, two GGPP molecules are then condensed to the colorless phytoene by a phytoene synthase. Phytoene is processed to the red-colored lycopene, which in turn is converted to β-carotene by a lycopene cyclase, which catalyzes the formation of β-ionone rings on the chain ends (Fig. 1). And third, further stepwise hydroxylations and ketolations of the ionone rings of β-carotene lead to the biosynthesis of various xanthophylls, including zeaxanthin, echinenone, canthaxanthin, and astaxanthin (Fig. 1).

Regarding heterologous ketocarotenoid production, only two β-carotene ketolases have been tested in cyanobacteria: CrtW, the β-carotene oxygenase from the proteobacterium *Brevundimonas* sp. SD-212 and HpBKT, the β-carotene ketolase from *H. pluvialis*. Therefore, in this study, we aimed to explore other pathways to ketocarotenoid production, with a focus on astaxanthin. To this end, we first tested the CrtR/CrtS system of *X. dendrorhous*. CrtS is a P450 astaxanthin synthase, and CrtR has been identified as the corresponding P450 reductase (CPR) for the biosynthesis of astaxanthin from β-carotene (Alcaíno et al. 2008). The expression of eukaryotic cytochrome P450 (hereafter P450) enzymes remains a challenge in traditional heterotrophic hosts. However, cyanobacteria have successfully been used on multiple occasions to express eukaryotic P450s (Lassen et al. 2014, Berepiki et al. 2016, 2018, Wlodarczyk et al. 2016, Santos-Merino et al. 2021, Torrado et al. 2022, Sutardja et al. 2024, Hanamghar et al. 2025). Second, we tested the β-carotene ketolase from *Chlamydomonas reinhardtii*, CrBKT, which has been reported multiple times to efficiently produce astaxanthin. In *C. reinhardtii*, the *CrBKT* gene has very low expression levels and is considered not active (Lohr et al. 2005). Synthetic redesign of the gene led to astaxanthin production in *C. reinhardtii* (Perozeni et al. 2020). Heterologous expression of *CrBKT* enabled astaxanthin production in *Arabidopsis* (Zhong et al. 2011), tomato (Huang et al. 2013), *Nannochloropsis oceanica* (Canini et al. 2024), and *Escherichia coli* (Lu et al. 2017, Park et al. 2018).

Ultimately, we show that expression of CrBKT is sufficient to achieve the production of canthaxanthin and astaxanthin in cyanobacteria and that this enzyme will likely play a key role in the future of sustainable ketocarotenoid production.

## Materials and methods

### Strains and growth conditions

A glucose-tolerant, non-motile strain *Synechocystis* sp. PCC 6803 “Kaplan” (hereafter Syn6803) and the fast-growing strain *Synechococcus elongatus* UTEX 2973 (Yu et al. 2015) (hereafter Se2973) were used in this study. Cyanobacterial strains were cryo-preserved in 15% glycerol and regularly taken out of cryostocks on agar plates for experiments. Active cyanobacterial cultures were maintained at 30°C with 10–50 μmol photons m^−2^ s^−1^ white light on 1.5% (w/v) agar plates of BG-11 medium (Stanier et al. 1971) supplemented with 10 mM *N*-Tris(hydroxymethyl)methyl-2-amino-ethanesulphonic acid buffer (pH 8.0) and 3 g l^–1^ sodium thiosulfate pentahydrate. For transgenic strains, the growth medium was also supplemented with appropriate antibiotics at a concentration of 50 µg ml^–1^ kanamycin (Crt strains) or spectinomycin (BKT strains).

Liquid cultures were grown in phosphate replete (P4) medium (Lippi et al. 2018) bubbled with 3% CO_2_ (v/v) supplemented air in glass tubes in a Multicultivator MC1000 (Photon Systems Instruments). Kanamycin (Crt strains) or spectinomycin (BKT strains) was added at a concentration of 50 µg ml^–1^ to the liquid cultures. Syn6803 cultures were incubated at 30°C and illuminated with ~50 µmol photons m^−2^ s^−1^, and Se2973 cultures were incubated at 38°C with ~100 µmol photons m^−2^ s^−1^ unless stated otherwise. For growth analysis, cultures were inoculated from liquid precultures to an OD_750 nm_ = 0.3 and induced with 0.1 mM isopropyl β-D-1-thiogalactopyranoside. Se2973 was grown at 38°C with a linear light gradient from 250 to 900 µmol photons m^−2^ s^−1^ in 18 h followed by constant illumination at 900 µmol photons m^−2^ s^−1^. Syn6803 strains were grown at 30°C with a linear light gradient from 30 to 100 µmol photons m^−2^ s^−1^ in 72 h followed by constant illumination at 100 µmol photons m^−2^ s^−1^.

To determine cell dry weight (CDW), 10 ml of cultures were transferred into pre-weighed 15 ml centrifuge tubes and centrifuged at 3200 × *g* for 15 min. All cell pellets were then washed, lyophilized for 24 h, allowed to equilibrate to room temperature, and weighed.

### Generation of constructs

All cloning and plasmid amplification was performed in *E. coli* NEB 5-alpha (New England Biolabs). An overview of all plasmids used in this study is shown in Table 1. Primers used for insert amplification are listed in Table S1. The insert sequence of all generated constructs was verified by Sanger sequencing. Plasmid maps of all constructs generated in this study were deposited in Zenodo (DOI: 10.5281/zenodo.14724050).

**Table 1.** Plasmids used in this study.

Plasmid	Details	Reference	Strains generated using the respective plasmid
pJZ66	Integrative plasmid for integration at neutral site (NS) *slr1068* (Syn6803) with *lacI*, Ptrc and T_BBa_B0015_; selection marker: Kanamycin^R^	This study, modified from pEERM3 (Englund et al. 2015)	N^Kan^
pJZ106	Integrative plasmid with *crtS*-*HA*; backbone: pJZ66	This study	CrtS
pJZ127	Integrative plasmid with *crtS*-*HA, crtR-HA*; backbone: pJZ66	This study	CrtSR
pJZ144	Integrative plasmid with *crtS*-*HA, crtR:A2_G19del-HA*; backbone: pJZ66	This study	CrtSR^∆^
pDF-trc	Self-replicating plasmid, pTrc, TrrnB T1 + TrrnB T2, Selection Marker: Spectinomycin^R^/Streptomycin^R^ (*aadA*)	Guerrero et al. (2012)	Syn6803-N^Spec^, Se2973-N^Spec^
pJZ160	Self-replicating plasmid with *CrBKT-FLAG*, backbone: pDF-trc	This study	Syn6803-BKT, Se2973-BKT

For expression of the *crtS* and *crtR* genes in Syn6803, first a general acceptor backbone was generated for genome integration targeting the neutral site *slr1068* on the Syn6803 chromosome. To achieve this, the plasmid pEERM3 was kindly provided by Pia Lindberg and used as a backbone (Addgene plasmid # 64 025, Englund et al. 2015). The nrsB promoter was exchanged with a *lacI* cassette and a trc promoter as encoded on the plasmid pDF-trc (Guerrero et al. 2012) using restriction digest cloning keeping the multiple cloning site intact. The generated acceptor plasmid was termed pJZ66. The target genes were then consecutively inserted using a biobrick-like method of restriction digest cloning with the enzymes XbaI and SpeI (Englund et al. 2015). A DNA fragment encoding a C-terminal HA-tag was added by PCR together with the restriction sites XbaI and SpeI for cloning purposes to all genes of interest. The gene encoding the astaxanthin synthase CrtS from *X. dendrorhous* (UniProt ID Q3HR17) was inserted first, and this construct was termed pJZ106. Two further constructs were generated using pJZ106 as a backbone to generate a synthetic operon of c*rtS* followed by the gene encoding the CPR CrtR (UniProt ID B6D970). The *crtR* gene was either inserted without any modification retaining the native ER-targeting signal peptide (plasmid termed pJZ127) or without the fragment encoding the native N-terminal signal peptide (AA 1-19) generating the plasmid termed pJZ144.

For expression of the β-carotene ketolase encoding gene from *C. reinhardtii* (*CrBKT*) in cyanobacteria, the full-length amino acid sequence of CrBKT (UniProt ID: Q4VKB4, GenBank: AY860820.1) fused to a C-terminal FLAG-tag was codon-optimized for expression in Syn6803 using the software Gene Designer 2.0 (Villalobos et al. 2006). The optimized DNA sequence was custom-synthesized by GenScript (USA). The *CrBKT-FLAG* gene was then inserted into the shuttle vector pDF-trc (Guerrero et al. 2012) by restriction digestion and ligation using the restriction sites EcoRI and HindIII. The restriction sites were added to the *CrBKT-FLAG* fragment by PCR. Vector and insert fragments were ligated using T4 DNA Ligase (New England Biolabs) to generate the final plasmid named pJZ160.

### Strain generation and colony PCR of cyanobacteria

For genome integration of constructs in Syn6803, a standard protocol of natural transformation was used. In brief, cells were grown to mid-exponential growth in BG-11 medium. Cells of a 10 ml culture grown in BG-11 medium were then concentrated by centrifugation and resuspended in fresh BG-11 medium and mixed with 1 µg of purified target plasmid DNA. The DNA-cell mixture was incubated overnight at 30°C in the dark and plated the next day on BG-11 medium agar plates without the supplementation of antibiotics followed by a 1 d incubation in low light conditions at 30°C. The cells were then carefully washed off the plate with fresh liquid medium and plated on BG-11 medium agar plates supplemented with 10 µg ml^−1^ kanamycin and incubated at 30°C with ~50 µmol photons m^2^ s^−1^ illumination until colonies appeared. Single colonies were re-streaked on increasing concentrations of kanamycin-supplemented BG-11 plates up to 50 µg ml^−1^ for segregation. Fully segregated transformants were confirmed by colony PCR using the primers slr0338-F (5′-GCC CTC AGT GCG AGT GTT TAA-3′) and slr0169-R (5′-TAC GGA GTT CGA TGC CCA T-3′).

For transfer of shuttle vector constructs (pDF-trc and pJZ160) into Syn6803 or Se2973, a standard protocol for biparental mating was used as previously described (Russo et al. 2019). Colony PCR was performed to confirm the presence of the plasmid using the primers trc-F and trc-R (Russo et al. 2019).

### Preparation of cell lysates

Cleared cell lysates were prepared as previously described (Russo et al. 2019). For cell lysates directly subjected to SDS-PAGE (sodium dodecyl sulfate–polyacrylamide gel electrophoresis), a modified lysis buffer consisting of 20 mM Tris–HCl pH 7.5, 5% glycerol, and 0.5% ASB-14 (Serva Electrophoresis GmbH) was used. Lysates for cell fractionation assays were prepared using the same method without adding any detergent to the lysis buffer.

### Cell fractionation assays

For cellular fractionations, first cell lysates without the addition of a detergent were prepared. These lysates were then directly used for a cellular fractionation protocol using ultracentrifugation and sucrose gradients as previously described (Russo et al. 2019). Using this cellular fractionation protocol, three protein fractions were separated: a soluble, a total membrane, and an enriched thylakoid membrane fraction. All fractions and the unprocessed lysate samples were then analyzed using SDS-PAGE and immunoblotting.

### SDS-PAGE and immunoblotting

Protein content of samples was estimated using a bicinchoninic acid protein assay (Thermo Fisher Scientific). For analysis of cleared cell lysates, samples were processed as described elsewhere (Russo et al. 2022), and 10 µg of protein were loaded per sample. For the analysis of cellular fractions, 20 µg of protein were loaded per sample. SDS-PAGE and immunoblotting were performed following previously established protocols as described in Russo et al. (2019).

### Pigment extraction and absorbance measurements

All absorbance measurements were carried out on a Synergy H4 Hybrid Reader (BioTek). For absorbance spectra, absorbance was measured from 400 to 750 nm in 1 nm steps. Pigments were extracted from cyanobacterial cultures using ice-cold methanol as previously described (Zavřel et al. 2015, Russo et al. 2019). The pigment content was then calculated using previously described equations for chlorophyll *a* (Ritchie 2006) and total carotenoid content (Wellburn 1994). For estimation of phycocyanin content, cultures were directly used for absorbance measurements without further treatment. The phycocyanin content was then calculated as described in Myers et al. (1980).

### Thin layer chromatography

For high-performance thin-layer chromatography (HPTLC) analysis, a protocol previously described was used (Dodge et al. 2020). In brief, pigment extracts were prepared in 100% ice-cold methanol (1 ml MeOH per OD _750 nm_ = 10 equivalents). A volume of 10 µl of pigment extracts were spotted on a TLC Silica gel 60 RP-18 F_254_ S plate (Supelco) and separated for 45 min using an acetone/methanol/water mixture (7:11:1, v/v/v) as mobile phase. As a positive control for the Syn6803 Crt strains, the pigment extract from the negative control strain N^Kan^ was spiked with 0.17 mM of a commercial astaxanthin standard (SML0982, Sigma-Aldrich).

### HPLC analysis of carotenoids

For HPLC analysis, cultures were grown in glass tubes with CO_2_-supplemented bubbling as detailed above. Cultures were harvested after 7 days of growth by centrifugation. The resulting pellets were then put under a nitrogen stream for 1 h, frozen at −80°C, and, subsequently, lyophilized. The dried pellets were mechanically ground to a fine powder with a glass rod. Approximately 5 mg of the powder was weighed into a glass vial. For ultrasonic extraction, 300 µl of a mixture of ethyl acetate/methanol (2:1, v/v) was added, and the samples were sonicated for 10 min. After centrifugation, the supernatant was transferred. The extraction process was repeated three times for each sample, and the collected supernatant was quantified gravimetrically, considering the residual mass of the initial pellet and residual extraction solution.

For HPLC analysis, a JASCO system (autosampler: AS-2055, pump: PU-2085, DAD-detector: MD-2010, AD-converter: LC-NetII/ADC, software: ChromNav 2.0) was used. A 2.1 × 100 mm C18 column (Purospher Star RP-C18e, Merck) was chosen and operated at a constant flow rate of 600 µl min^−1^. Column temperature was kept constant at 35°C for all runs. A gradient method was used for separation using eluent A (50% water, 22.5% methanol, 22.5% acetonitrile, 5% ethyl acetate, and 325 mg l^−1^ ammonium formate) and eluent B (50% methanol and 50% ethyl acetate) (Table S2). The samples were separated by a linear gradient from 30% to 82.5% B for 6.5 min, then to 100% B over 3.5 min and kept at 100% B for 2 min. Re-equilibration was then done by raising to 30% B over 0.5 min and holding at 30% B for a further 4 min. All eluents were degassed by sonication for ~15 min. All solvents, water, and ammonium formate were liquid chromatography–mass spectrometry (LC-MS) grade and purchased from a commercial supplier (Sigma-Aldrich). The following standards were used for method calibration: astaxanthin (SML0982, Sigma-Aldrich), zeaxanthin (orb702563, Biozol), canthaxanthin (trans, 32993, Sigma-Aldrich Supelco), and β-carotene (C4582, Sigma-Aldrich). Prodan (CAS: 70504-01-7) was used as an internal standard for calibration. Chromatograms (Fig. S1), UV spectra of standards (Fig. S2), and further details of calibration parameters (Table S3) can be found in the Supplementary Material.

## Results

### The CrtS enzyme from *Xanthophyllomyces dendrorhous* is not functional in cyanobacteria

**Figure 2.**
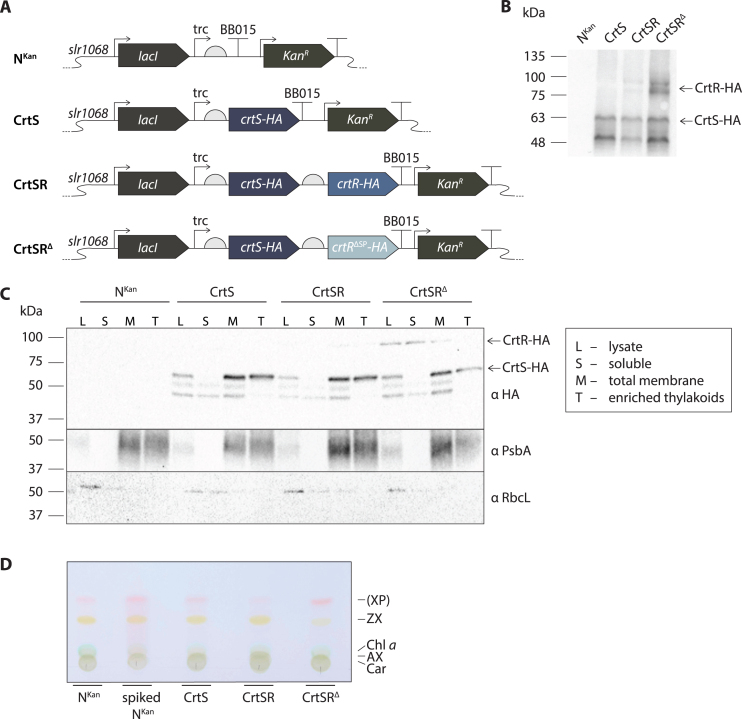
Production of fungal enzymes for ketocarotenoid production in engineered *Synechocystis* sp. PCC 6803. N^Kan^: negative control strain; CrtS: expression of *crtS* from the red yeast *Xanthophyllomyces dendrorhous*; CrtSR: co-expression of *crtS with crtR* from *X. dendrorhous*; CrtSR^∆^: co-expression of *crtS* with *crtR^∆^*. (AAs 1-19, which encode a predicted signal peptide, were removed.) All *crt* genes were fused with a sequence encoding a C-terminal HA-tag for detection purposes. (A) Overview of generated strains with constructs integrated at the *slr1068* locus. (B) Anti-HA immunoblot of whole cell lysates. For each sample, 10 µg of protein was loaded. (C) Subcellular fractions (L—lysate, S—soluble fraction, M—total membrane fraction, T—thylakoid enriched fraction) analyzed by immunoblotting using an HA antibody. Control blots for fractionations using an Anti-PsbA antibody and anti-RbcL are shown. In each lane, 20 µg of estimated protein of the respective fraction was loaded. (D) HPTLC of MeOH pigment extracts from respective strains (N^Kan^, CrtS, CrtSR, CrtSR^∆^). As a positive control, the negative control extract was spiked with 0.17 mM astaxanthin (spiked N^Kan^). Solid phase: 60 RP-18 F_254_ S; mobile phase: acetone/methanol/water (7:11:1, v/v/v); run time: 45 min. Major pigments are marked: Car—carotenes, AX—astaxanthin, Chl *a*—chlorophyll *a*, ZX—zeaxanthin, (XP): other unidentified xanthophylls.

Three constructs were designed for chromosomal insertion into the *slr1068* neutral site in Syn6803 (Fig. 2A): CrtS alone, CrtS with its partner CPR CrtR, and CrtS with a modified version of CrtR where the native signal peptide (which targets and anchors the protein to the endoplasmic reticulum membrane) was removed (hereafter CrtR^Δ^). Immunoblotting of whole cell lysates confirmed the expression of CrtS, CrtR, and CrtR^Δ^ at their expected molecular weights of 62.6, 81.9, and 79.9 kDa, respectively (Fig. 2B). For both proteins, a double-band pattern was observed with the lower molecular weight bands most likely representing products of unspecific cleavage. To further explore the cellular localization of CrtS, CrtR, and CrtR^Δ^, whole cells were fractionated into soluble, total membrane, and thylakoid-enriched fractions. Immunoblotting of the separate fractions revealed that CrtS was mostly localized to the membrane fraction with a large part of the protein detected in the thylakoid membranes (Fig. 2C). Curiously, in the soluble fraction only the suspected cleavage products of CrtS were found. In contrast, CrtS was found as a single band at its expected molecular weight in the thylakoid-enriched fraction. This suggests that CrtS is only stable when membrane embedded. In the CrtSR strain, CrtR was only visible as a faint band in the total membrane fraction. In the CrtSR^Δ^ strain, CrtR^Δ^ was clearly visible in the soluble fraction with only a minor band visible in the total membrane fraction (Fig. 2C). Overall, this suggests that removal of the native signal peptide improved expression but may have compromised membrane targeting. Given that CrtS, CrtR, and CrtR^Δ^ were expressed, the next step was to investigate whether the enzymes were active and producing astaxanthin. To this end, pigments were extracted and separated by HPTLC (Fig. 2D). However, pigment profiles of the Crt strains did not show differences compared to the WT control. Astaxanthin was only observed in the positive control (i.e. extracted pigments from the negative control spiked with astaxanthin) and was absent from all the engineered Crt Syn6803 strains. There was also no difference in growth observed in the CrtS/R-engineered strains compared to the negative control (Fig. S3).

### A microalgal β‐carotene ketolase is effective for ketocarotenoid production in cyanobacteria

**Figure 3.**
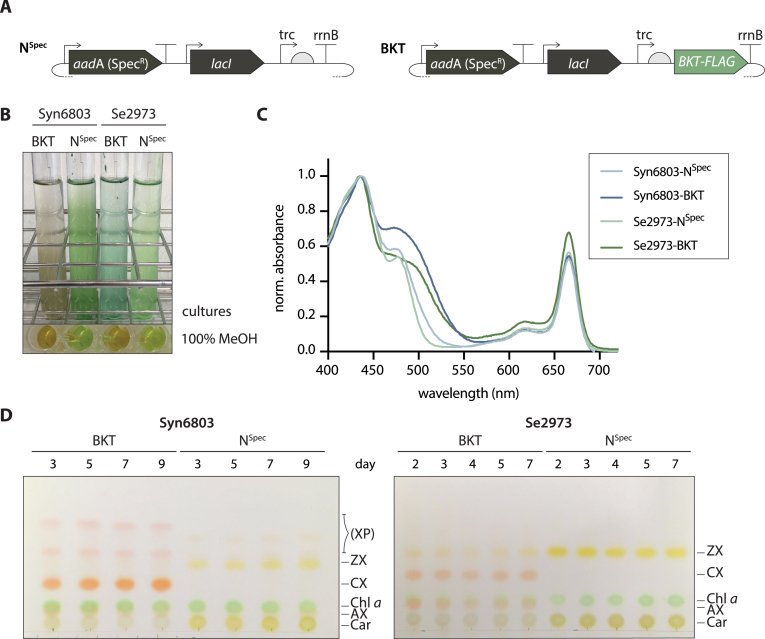
Activity assays of CrBKT in the cyanobacteria *Synechocystis* sp. PCC 6803 (Syn6803) and *Synechococcus elongatus* UTEX 2973 (Se2973). N^Spec^: negative control strain; BKT: expression of the microalgal β-carotene ketolase gene (*BKT)* from *Chlamydomonas reinhardtii* fused with a sequence encoding a C-terminal FLAG-tag. (A) Overview of construct design for plasmid-based *BKT* expression. (B) Color change in Syn6803 and Se2973 cultures expressing *BKT* and respective methanol (MeOH) pigment extracts. (C) Absorption spectra of MeOH pigment extracts from generated strains. (D) HPTLC of MeOH pigment extracts from selected cultivation timepoints. Solid phase: 60 RP-18 F_254_ S; mobile phase: acetone/methanol/water (7:11:1, v/v/v); run time: 45 min. Major pigments are marked: Car—carotenes, AX—astaxanthin, Chl *a*—chlorophyll *a*, CX—canthaxanthin, ZX—zeaxanthin, (XP): other unidentified xanthophylls.

Given the lack of astaxanthin production after engineering the *X. dendrorhous* CrtS P450 in Syn6803, we decided to test the *C. reinhardtii CrBKT*. To this end, a genetic construct was designed where the full-length *C. reinhardtii CrBKT* gene, fused to a FLAG-tag encoding sequence, was cloned into a shuttle plasmid under the control of the strong trc promoter (Fig. 3A). As a negative control, an empty vector control containing no gene of interest and an *aadA* selection marker cassette was used (Fig. 3A). These constructs were then conjugated into Syn6803. As it was not entirely clear whether the lack of ketocarotenoid production using the CrtS P450 in Syn6803 was due to enzyme/host incompatibility, the target plasmid was also used for conjugation into a different cyanobacterial host: the fast-growing *Synechococcus elongatus* UTEX 2973. Following growth and induction, a change of color was immediately visible in the strains harboring *CrBKT* (Fig. 3B). The Syn6803-BKT strain showed a brown color, while the Se2973-BKT culture had a visibly blue color in comparison to the negative control. Despite the different phenotypes in the BKT strains, the methanol pigment extracts both exhibited an orange-brown color, typical of ketocarotenoid overaccumulation (Fig. 3B). These differences were also evident in the absorption spectra of the BKT pigment extracts where a clear shoulder can be observed between 450 and 550 nm, the typical range for ketocarotenoid absorbance (Fig. 3C). For an initial determination of the pigment composition, pigment extracts of all four strains were separated by HPTLC. Using this method, the appearance of several distinct orange and red-colored ketocarotenoids in the BKT strains was clearly visible (Fig. 3D).

### Ketocarotenoid production leads to distinct metabolic changes in Syn6803 and Se2973

**Figure 4.**
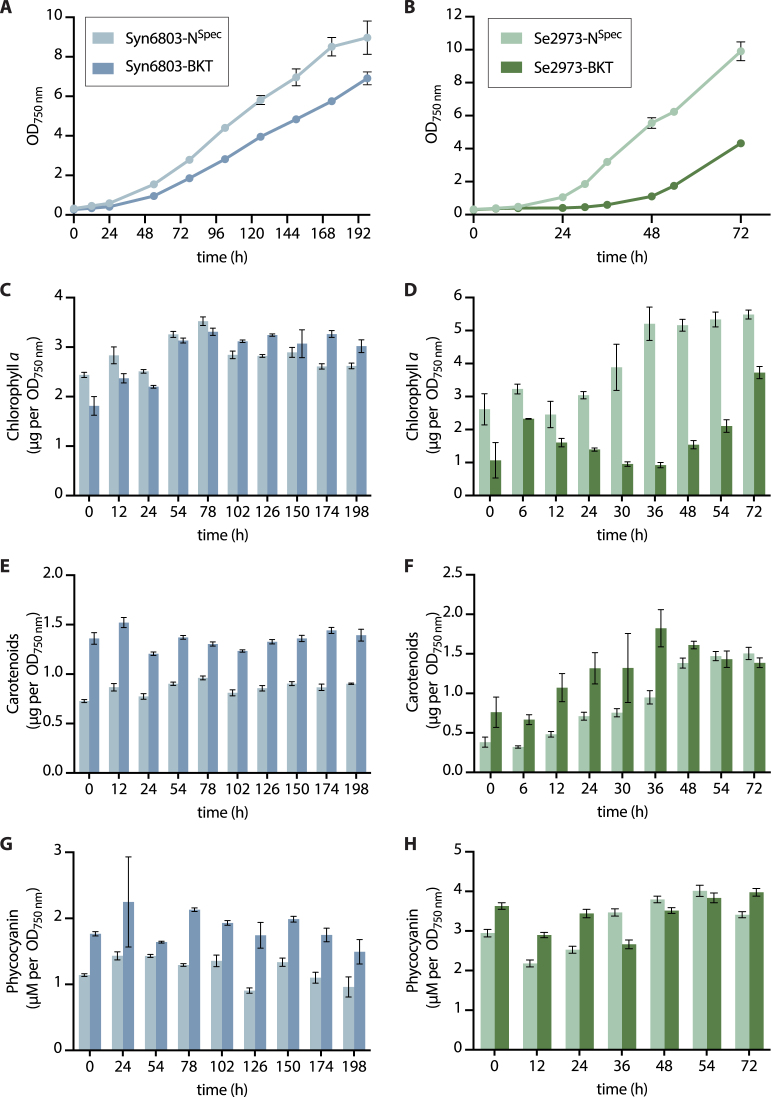
Growth and pigment analysis of BKT-producing *Synechocystis* sp. PCC 6803 (Syn6803) and *Synechococcus elongatus* UTEX 2973 (Se2973) strains. N^Spec^: negative control strain; BKT: *BKT-*expressing strain. (A, B) Growth monitored by measuring optical density at 750 nm (OD _750 nm_) with 3% CO_2_ supplemented by bubbling at (A) 30°C and light gradient from 30 to 100 µmol photons m^−2^ s^−1^ for Syn6803 and (B) 38°C with a light gradient from 250 to 900 µmol photons m^−2^ s^−1^ for Se2973. (C, D) Estimation of chlorophyll *a* in µg per OD_750 nm_, (E, F) total carotenoid content in µg per OD_750 nm_ and (G, H) phycocyanin levels in µM per OD_750 nm_ for Syn6803 and Se2973. *n* = 4, error bars: ±SD.

To further assess the impact of *CrBKT* expression on both cyanobacteria, the strains were grown in their respective ideal conditions (Cao et al. 2022), and pigment composition was analyzed over time. First, the growth curves showed that both BKT strains had a slower growth rate than their empty vector counterparts (Fig. 4A, B). This was particularly pronounced for Se2973-BKT, which only entered exponential growth at 48 h (Fig. 4B). Regarding biomass yields, Syn6803-BKT and Se2973-BKT achieved a final CDW of 1.16 and 1.18 g l^−1^, respectively. In comparison, Syn6803-N^spec^ and Se2973-N^spec^ achieved a final CDW of 1.51 and 2.59 g l^−1^, respectively (Fig. S4). Chlorophyll *a* levels in Syn6803-BKT did not exhibit any particular change in comparison to the empty vector control (Fig. 4C). However, in comparison to the empty vector control strain, Se2973-BKT exhibited a severe chlorophyll *a* deficiency with a loss of up to 82% at 36 h. The reasons for this chlorophyll deficiency are not yet clear; however, this is consistent with the astaxanthin-producing phenotype observed in *C. reinhardtii* (Perozeni et al. 2020). Chlorophyll *a* levels then recovered up to the end of the experiment at 72 h (Fig. 4D). This observation fits with the blue color that the Se2973-BKT strain exhibited in liquid culture (Fig. 3B). Regarding the levels of total carotenoids, BKT strains exhibited significantly higher concentrations than their respective empty vector controls (Fig. 4E, F). This is consistent with what was observed in the absorption spectra and the HPTLC analysis (Fig. 3C, D). It is worth noting that in the Se2973-BKT strain, the difference to the empty control was highest at 36 h (i.e. approximately double). After this timepoint, carotenoid concentrations between the two strains were similar up to the end of the experiment. Regarding phycocyanin levels, Syn6803-BKT exhibited consistently higher levels than its empty vector control (Fig. 4G). This trend was not visible in Se2973 where phycocyanin concentrations were not consistently higher in either strain over the course of the growth curve (Fig. 4H).

**Figure 5.**
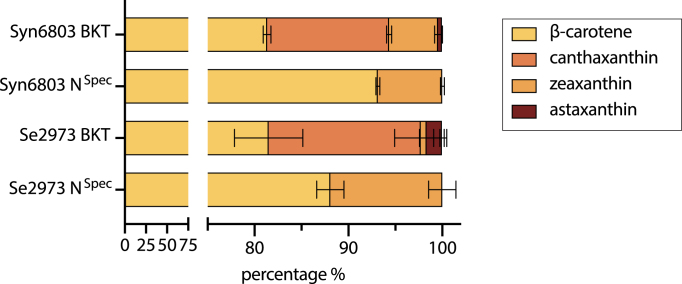
Relative percentage of selected carotenoids quantified by HPLC in *Synechocystis* sp. PCC 6803 (Syn6803) and *Synechococcus elongatus* UTEX 2973 (Se2973) strains. N^Spec^: negative control strain; BKT: *BKT*-expressing strain. *n* = 3, error bars: ±SD.

Finally, we sought to determine the concentration and distribution of the major carotenoids zeaxanthin, β-carotene, canthaxanthin, and astaxanthin. Therefore, HPLC analysis of pigment extracts from the generated strains was performed (Fig. S5). As expected, between the empty vector controls and the BKT strains, we observed a decrease in zeaxanthin and β-carotene with the appearance of the ketocarotenoids canthaxanthin and astaxanthin. In Syn6803-BKT, zeaxanthin and β-carotene were reduced by ~80% (from 0.2% to 0.04% of dry cell weight; DCW) and 77% (from 3% to 0.66% of DCW), respectively. This led to the production of canthaxanthin and astaxanthin constituting 13% and 0.4% of the measured carotenoids (Figs 5, S5). Curiously, in Se2973-BKT zeaxanthin and β-carotene were reduced by ~99% (from 0.7% to 0.004% of DCW) and 88% (from 5% to 0.6% of DCW), respectively. This led to the production of canthaxanthin and astaxanthin constituting 16% and 1.6% of the measured carotenoids (Fig. 5, S4). The stronger reduction in Se2973 zeaxanthin, in comparison to Syn6803, suggests the existence of differences in carotenoid metabolism between these cyanobacteria. Ultimately, canthaxanthin and astaxanthin amounted to 0.1% and 0.004% of DCW, respectively, in Syn6803-BKT. This corresponded to an astaxanthin yield of 0.024 mg l^−1^ and daily productivity of 0.003 mg l^−1^ d^−1^. In Se2973-BKT, canthaxanthin and astaxanthin amounted to 0.12% and 0.012% of DCW, respectively. This corresponded to an astaxanthin yield of 0.08 mg l^−1^ and daily productivity of 0.02 mg l^−1^ d^−1^.

## Discussion

In this study, we tested two eukaryotic paths to ketocarotenoid biosynthesis in cyanobacteria. We showed that while the astaxanthin synthase CrtS from the red yeast *X. dendrorhous* was not functional in Syn6803, the CrBKT from the green alga *C. reinhardtii* is a promising route for ketocarotenoid production in cyanobacteria.

Based solely on our data, it is not immediately obvious why CrtS was not functional; however, one can speculate. In all constructs, CrtS was only produced at the expected molecular weight when localized in, or at, the thylakoid membrane. The observation of CrtS degradation products in non-membrane fractions is not surprising given the documented instability of P450s outside membranous environments (Biggs et al. 2016). Despite correct expression and localization, CrtS alone was not sufficient for astaxanthin production. This is in agreement with previous studies that have shown that CrtS requires the very specific redox partner CrtR for activity (Alcaíno et al. 2008). However, in our work, co-expression of *crtR* together with *crtS* also did not result in the production of astaxanthin. Use of the full-length CrtR resulted in low protein yields, possibly explained by incompatibility of the native signal peptide/membrane anchor, which targets CrtR to the endoplasmic reticulum. Removal of this N-terminal sequence significantly improved expression levels; however, it also led to most of the protein being found in the soluble fraction. It has been suggested that CrtS and CrtR form a complex at the membrane surface (Alcaíno et al. 2012); therefore, it is plausible that the loss of membrane targeting of CrtR would have led to an inactive CrtS. In the future, fusion of CrtR with native thylakoid-targeting sequences could help with the membrane co-localization of the CrtS/CrtR complex. Finally, establishment of CrtS in *E. coli* was also not successful (Verwaal et al. 2007); therefore, we cannot exclude the requirement of specific posttranslational modifications not present in prokaryotic hosts.

The route to ketocarotenoid production that we presented in this study via the CrBKT enzyme was functional. However, it presented low yields in comparison to other green hosts expressing only *CrBKT* (Table 2). The maximum yields of canthaxanthin and astaxanthin obtained in this study were 0.12% and 0.01% of DCW, respectively, in Se2973-BKT, with astaxanthin volumetric yields of 0.08 mg l^−1^ and 0.02 mg l^−1^ d^−1^. In comparison, a *N. oceanica CrBKT* expression strain accumulated 0.2% and 0.07% of DCW for canthaxanthin and astaxanthin, respectively, with astaxanthin volumetric yields of 0.64 mg l^−1^ and 0.06 mg l^−1^ d^−1^ (Canini et al. 2024). The highest levels achieved in a phototrophic chassis in autotrophic conditions, without further engineering, were in a *C. reinhardtii* CrBKT strain, which accumulated up to 1 mg l^−1^ d^−1^ of astaxanthin (Perozeni et al. 2020). There are multiple reasons that could explain the lower yields in cyanobacteria reported here. First, both engineered cyanobacterial strains, Syn6803-BKT and Se2973-BKT, exhibited clear growth defects and were unable to achieve high cell densities (Fig. 4A, B). This was particularly evident for Se2973-BKT. In this strain, the lag phase was delayed until ~36 h. After 36 h, we then observed increasing chlorophyll concentrations and decreasing carotenoid concentrations (Fig. 4D, F). Therefore, it is possible that exponential growth only started when changes in *CrBKT* expression (e.g. accumulation of point mutations, plasmid loss) occurred. Second, the expression of CrBKT alone is likely not sufficient to produce astaxanthin in cyanobacteria. The key difference to microalgal hosts is that *C. reinhardtii* and *N. oceanica* natively possess hydroxylases capable of converting canthaxanthin to astaxanthin. On the other hand, the required hydroxylase activity is either insufficient or not present in cyanobacteria (Harker and Hirschberg 1997, Liang et al. 2023). A study published while this work was under review supports this idea. Expression of CrBKT alone in the fast-growing *Synechococcus* sp. PCC 11901 produced similar pigment profiles to our study (Fig. 3C). The co-expression of a β-carotene hydroxylase then led to a significant accumulation of astaxanthin up to 38.4 mg l^−1^ in over 4 days, the highest ever reported for a cyanobacterial host (Betterle et al. 2025).

**Table 2.** Comparison of heterologous production titers of the ketocarotenoid astaxanthin in microbial chassis. Yields shown in mg l^–1^, mg l^–1^ d^–1^, and mg g^–1^ CDW (cell dry weight) represent studies undertaken in phototrophic batch cultivation. Experiments performed under high light (>1000 µmol photons m^2^ s^−1^) have been excluded.

Organism	mg l^–1^	mg l^–1^ d^–1^	mg g^−1^ CDW	Reference
*Synechocystis* sp. PCC 6803	0.024	0.003	0.03	This study
*Synechococcus elongatus* UTEX 2973	0.078	0.02	0.12	This study
*Synechocystis* sp. PCC 6803	28	2.8	29.6	Diao et al. (2020)
*Chlamydomonas reinhardtii*	8.6	2.87	4.5	Amendola et al. (2023)
*Chlamydomonas reinhardtii*	0.0002	0.00 003	0.002	Perozeni et al. (2020)
*Chlamydomonas reinhardtii*	0.0003	0.00 005	0.002	Perozeni et al. (2020)
*Nannochloropsis oceanica*	0.64	0.06	0.7	Canini et al. (2024)
*Nannochloropsis oceanica*	68.1	9.9	7.3	Liu et al. (2024)
*Synechococcus* sp. PCC 11 901	24.7	6.18	5.6	Betterle et al. (2025)
*Saccharomyces cerevisiae* ^a^	55.7	16.88	10.1	Jin et al. (2018)
*Escherichia coli* ^a^	62	–	15.1	Zhang et al. (2018)

a Selected values from heterotrophic production chassis for comparison.

Beyond the expression of an appropriate hydroxylase, other strategies have also been employed in phototropic hosts to increase the production of astaxanthin (Table 2). For example, engineering of central carbon metabolism by overexpression of fructose-1,6-/sedoheptulose 1,7-bisphosphatase (F/SBPase) significantly increased astaxanthin production in Syn6803. Yields were then further increased, to a final 28 mg l^−1^, by engineering the methylerythritol phosphate (MEP) pathway to increase the available pool of β-carotene. This was achieved by co-producing a heterologous 1-deoxy-D-xylulose 5-phosphate synthase and farnesyl diphosphate synthase (IspA) and supplementation of 5 mM sodium pyruvate (Diao et al. 2020). A similar strategy of engineering the MEP pathway was employed in *N. oceanica*. Here, the native GGPP synthase-encoding gene was overexpressed to increase the GGPP pool. Together with the expression of an astaxanthin-binding protein, this study achieved a final yield of 68 mg l^−1^ in fed-batch cultivation (Liu et al. 2024). Engineering targets downstream of the MEP pathway also exist. For instance, a study in *C. reinhardtii* showed that the overexpression of the native phytoene synthase significantly increased astaxanthin yields (Amendola et al. 2023).

It is also worth mentioning that any metabolic engineering approach should be combined with a synthetic biology strategy to balance intermediate levels and metabolic burden according to the specific host. In this study, we observed that BKT expression had a significant impact on growth of Se2973-BKT, likely due to excessive metabolic burden. One option to mitigate this and lower the apparent metabolic burden would be to exchange the trc promoter for a weaker constitutive promoter or a tightly regulated, inducible promoter (e.g. by including a riboswitch). In the course of this study, we also designed and tested an inducible system based on the rhamnose promoter (Kelly et al. 2018, Behle et al. 2020). However, no protein expression or changes in pigment composition were observed (data not shown).

In conclusion, we have shown that CrBKT is a promising option for astaxanthin production in cyanobacteria. High yields will likely require balancing promoter strength, screening for partner β-carotene hydroxylases, and further optimization of growth conditions. These improvements can also be coupled with metabolic engineering strategies to increase precursor pools. Considering their ease of engineering, fast photoautotrophic growth, and high biomass yields, cyanobacteria could become a sustainable source of astaxanthin capable of displacing *H. pluvialis*.

## Supplementary Material

qvaf008_Supplemental_File

## Data Availability

The data underlying this article are available in the article and in its online supplementary material. Sequences of plasmids generated in this study are available in Zenodo and can be accessed with DOI: 10.5281/zenodo.14724050. All other materials are available from the corresponding authors upon reasonable request.
