# Supplementary material for: Testing eukaryotic routes for heterologous production of ketocarotenoids in cyanobacteria
Source: Sustain Microbiol. 2025 Jun 3;2(2):qvaf008. doi: 10.1093/sumbio/qvaf008 (PMC13375318; doi:10.1093/sumbio/qvaf008)
Supplement: Supplementary file 1 [file qvaf008_Supplemental_File.pdf]

Supplementary Data

Testing eukaryotic routes for heterologous production of ketocarotenoids in cyanobacteria

Anika V. Mahdi[1], Dennis N. Wittmann[1,a], Sayali S. Hanamghar[1], Oskar Zorć[1,b], Jialan Cao[2], G. Alexander Groß[2], Poul Erik Jensen[3], David A. Russo[4] and Julie A. Z. Zedler[1*]*

[1] Synthetic Biology of Photosynthetic Organisms, Matthias Schleiden Institute for Genetics, Bioinformatics and Molecular Botany, Friedrich Schiller University Jena, 07743 Jena, Germany
[2] Department of Physical Chemistry and Microreaction Technology, Institute of Chemistry and Bioengineering, Ilmenau University of Technology, 98693 Ilmenau, Germany
[3] Department of Food Science, University of Copenhagen, 1958 Frederiksberg, Denmark
[4] Bioorganic Analytics, Institute for Inorganic and Analytical Chemistry, Friedrich Schiller University Jena, 07743 Jena, Germany
[a] Present address: STADA Arzneimittel AG, Stadastraße 2-18, 61118 Bad Vilbel
[b] Present address: Bio Pilot Plant, Leibniz Institute for Natural Product Research and Infection Biology - Hans Knöll Institute, 07745 Jena, Germany

[*]Correspondence: david.russo@uni-jena.de, julie.zedler@uni-jena.de

Figure S1 Chromatogram traces of HPLC standards.

Figure S2 UV-spectra of analyzed carotenoids.

Figure S3 Growth analysis of *Synechocystis* sp. PCC 6803 transformants producing the fungal astaxanthin synthase CrtS.

Figure S4 Calibration Curves of $OD_{750\ nm}$ vs. cell dry weight for Syn6803-BKT, Se2973-BKT strains and respective negative controls.

Figure S5 HPLC chromatograms of Syn6803-BKT, Se2973-BKT strains and respective negative controls.

Table S1 Primers used for plasmid generation.

Table S2 HPLC-Gradient used in this study.

Table S3 Chromatographic parameters for the quantification of the target compounds.

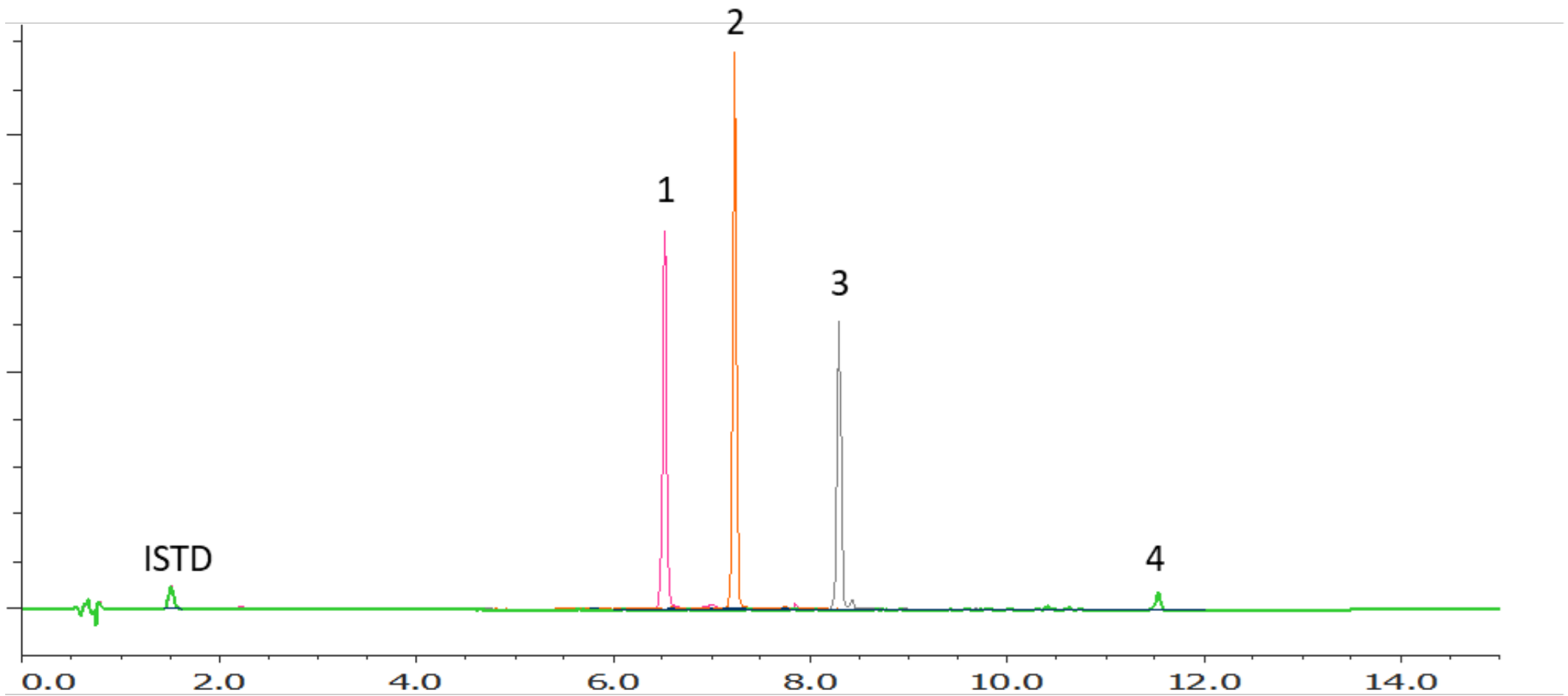

Figure S1. Chromatogram traces of the target compounds at λ_{max}. Absolute reference masses: Astaxanthin (1) 39 ng; Zeaxanthin (2) 88 ng; Canthaxanthin (3) 14 ng, β-Carotene (4) 584 ng. ISTD: internal standard.

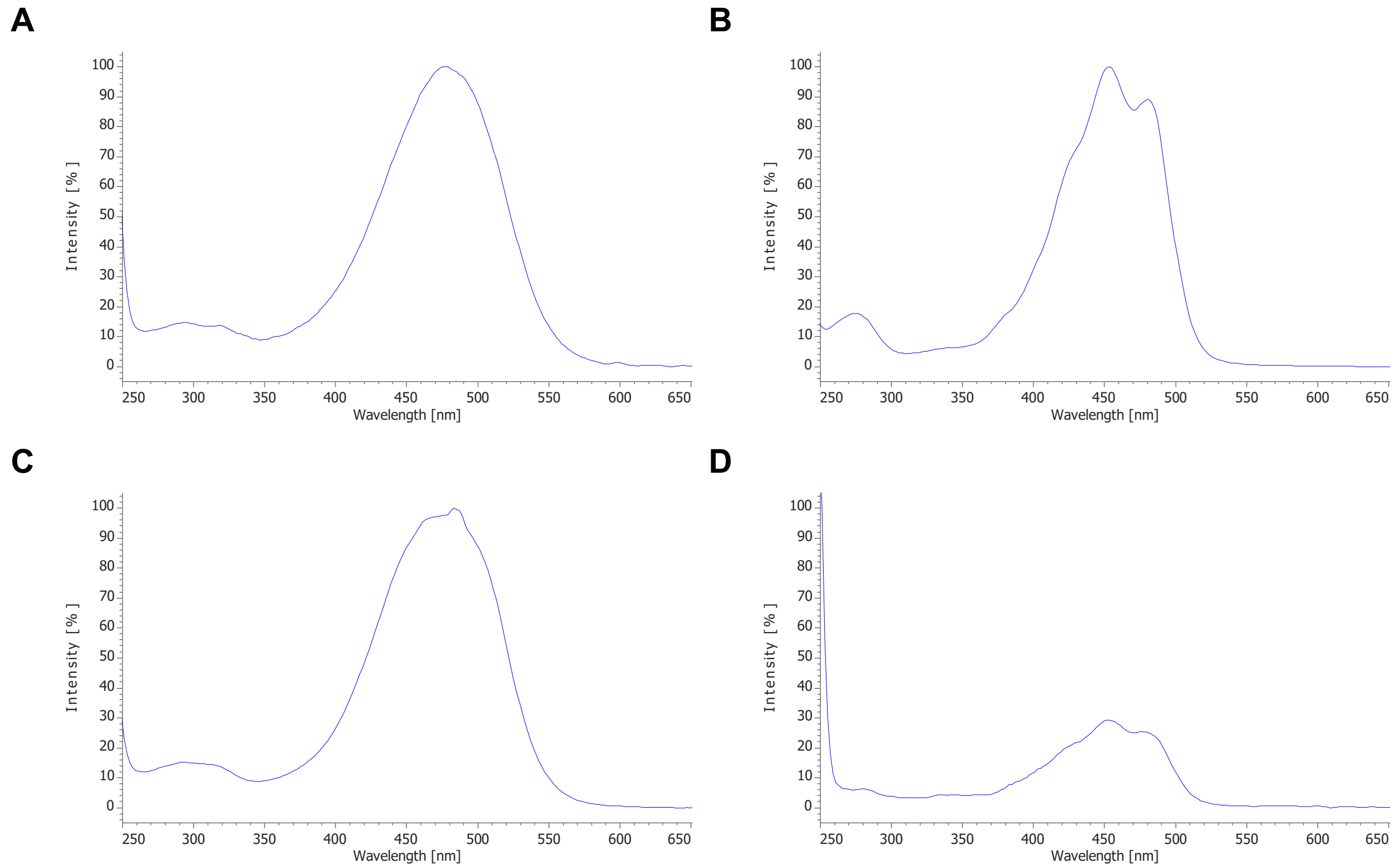

Figure S2. UV-Spectra of analyzed carotenoids: (**A**) Astaxanthin (**B**) Zeaxanthin **(C)** Canthaxanthin, (**D**) β-Carotene.

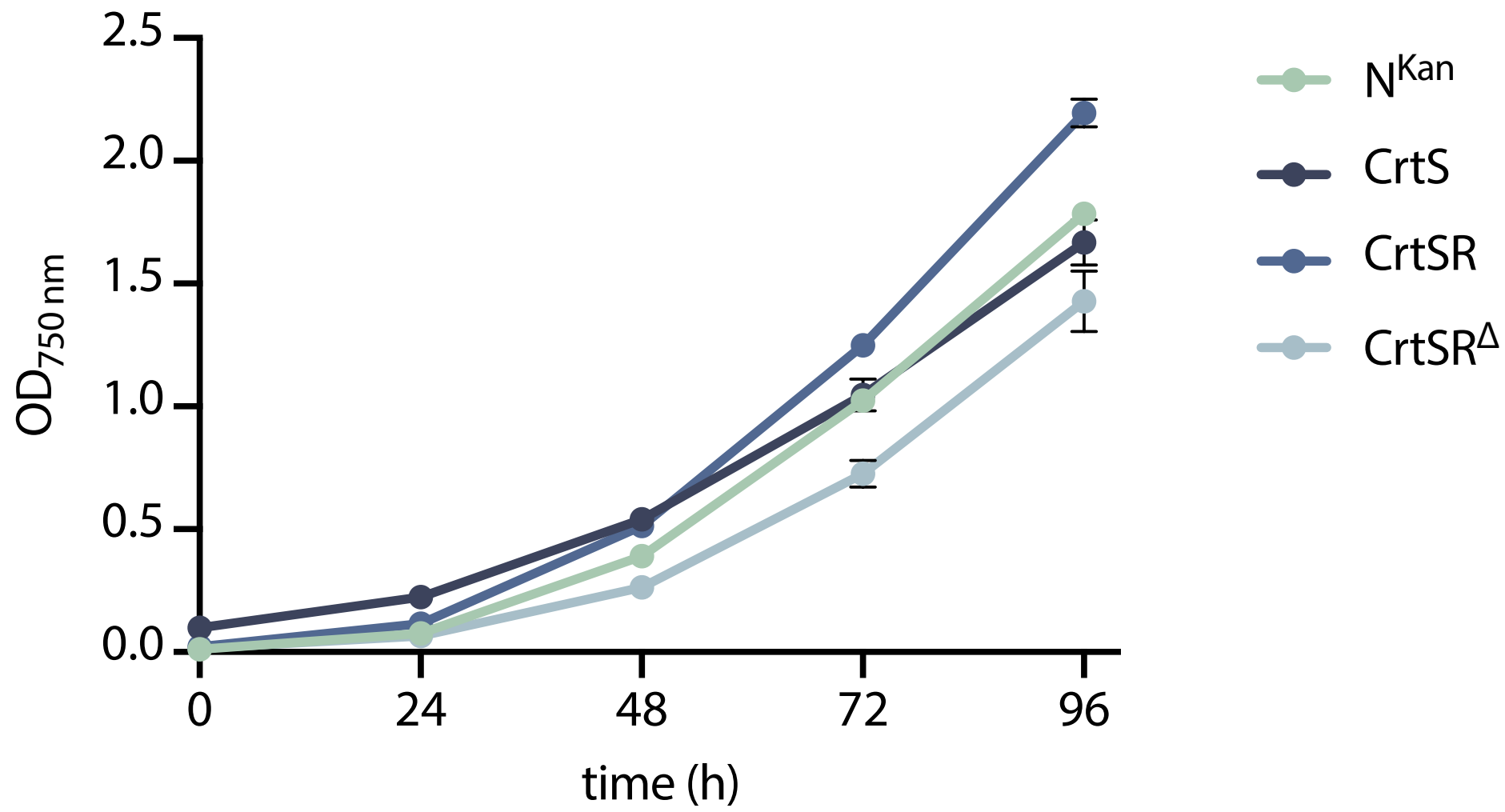

Figure S3. Growth analysis of *Synechocystis* sp. PCC 6803 transformants producing the fungal astaxanthin synthase CrtS. N^{Kan}: negative control strain; CrtS: Expression of *crtS* from the red yeast *Xanthophyllomyces dendrorhous*; CrtSR: Co-expression of *crtS with crtR* from *X. dendrorhous*; $CrtSR^{\Delta}$: Co-expression of *crtS* with *crtR*$^{\Delta}$. Growth monitored by measuring optical density at 750 nm ($OD_{750\ nm}$) with 3% CO_2 supplemented by bubbling at 30°C with constant illumination at 100 µmol photons m^{-2} s^{-1}. Cultures were induced with 1 mM IPTG at inoculation, n=3, error bars: ±SD.

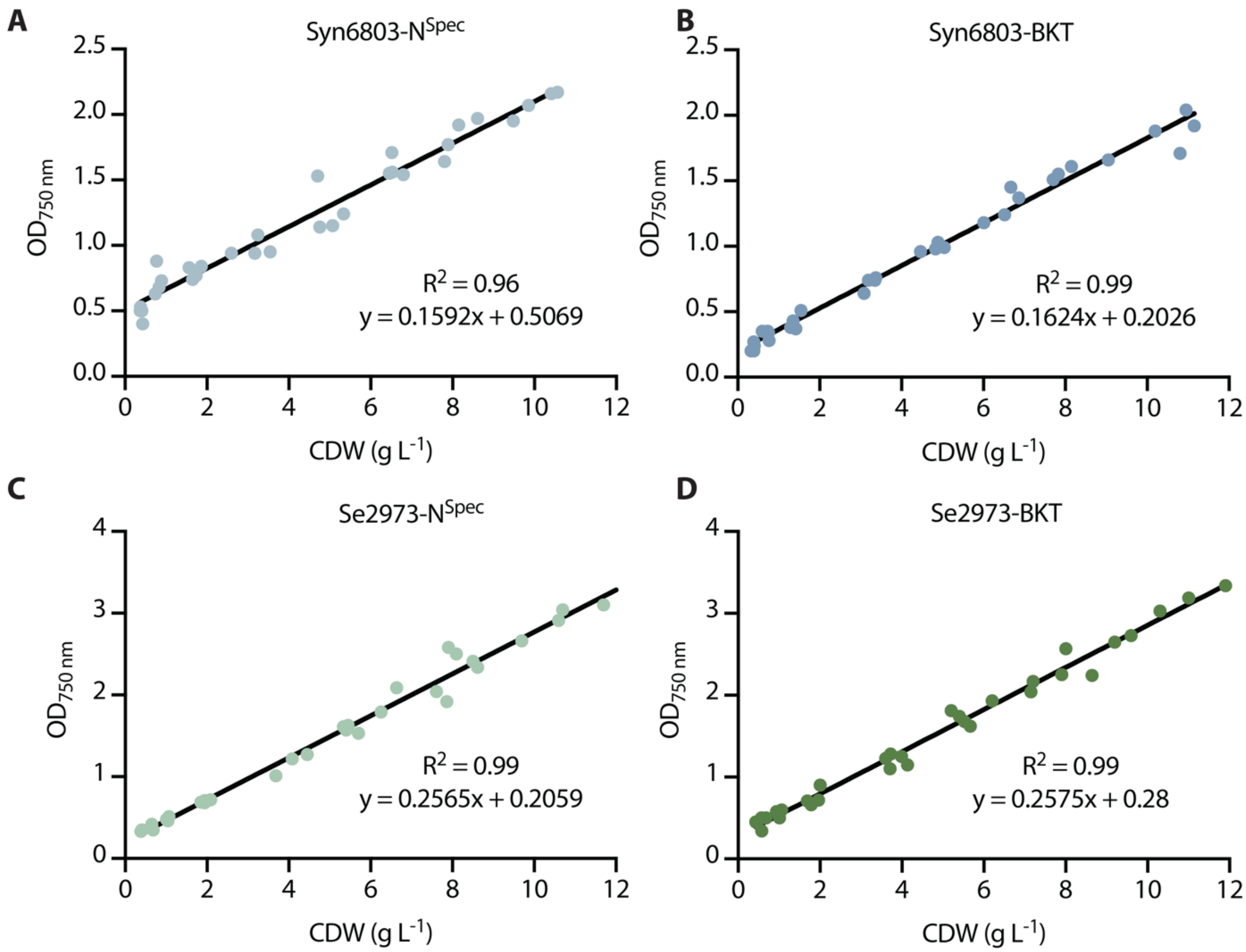

Figure S4. Correlation between $OD_{750\ nm}$ and cell dry weight in *Synechocystis* sp. PCC 6803 (Syn6803) and *Synechococcus elongatus* UTEX 2973 (Se2973) strains. Calibration curves are shown for the strains **A** Syn6803-N^{Spec}, **B** Syn6803-BKT, **C** Se2973-N^{Spec} and **D** Se2973-BKT, n=4 for all strains.

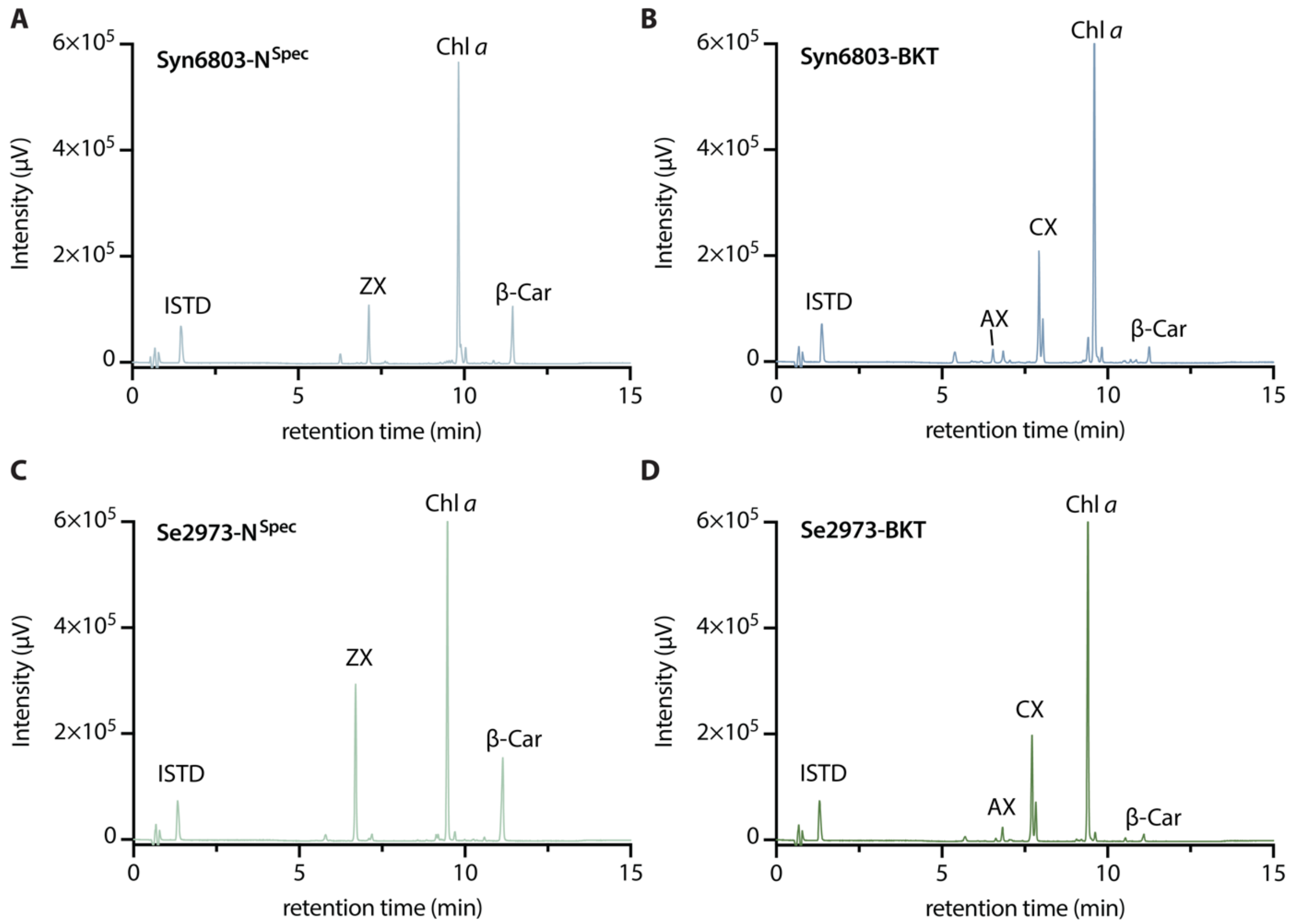

Figure S5. HPLC Chromatograms of pigment extracts in ethyl acetate/methanol (2:1, v/v) extracted from dried biomass of **A, B** *Synechocystis* sp. PCC 6803 (Syn6803) and **C, D** *Synechococcus elongatus* UTEX 2973 (Se2973) strains. N^{Spec}: negative control strains (B,D); BKT: *BKT* expressing strain (A, C). Peaks of major pigments identified are labelled in all panels, AX – astaxanthin, β-Car – β-carotene, Chl *a* – chlorophyll *a*, CX – canthaxanthin, ZX – zeaxanthin, ISTD: internal standard.

Table S1 Primers used for plasmid generation.

Primer Name	Sequence (5'-3')	Purpose
Waldo1F	TGT GCC TCT AGA ATG TTC ATT CTG GTT CTG CTG ACG GG	Generation of *crtS-HA* insert for pJZ106 plasmid
Waldo1R	AAT TAC ACT AGT CTA TTA GGC GTA GTC TGG CAC ATC GTA GGG ATA TTC CAC CGG CTT CAC TTG CA	
Waldo2F	TGT GCC TCT AGA ATT AAA GAG GAG AAT ACT AGA TGG CAA CGC TGT CGG ACC T	Generation of *crtR-HA* insert for pJZ127 plasmid
Waldo2R	GAT TAC ACT AGT CTA TTA TGC GTA GTC GGG CAC GTC ATA GGG GTA CGA CCA GAC ATC CAT CAG CAG A	
Waldo 3F	TGT GCC TCT AGA ATT AAA GAG GAG AAT ACT AGA TGT TCT ACA ACA AAG ACC GCC TG	Generation of *crtRΔSP-HA* insert for pJZ144 plasmid (+rev primer: Waldo2R)
EcoRI_OvBKT_F	TAA GAA TTC ATG GGG CCC GGC ATC CAA CC	Generation of *CrBKT-FLAG* insert for pJZ160 plasmid
2.0 BKT-HindIII_OvR	TAT AAG CTT CTA CTA GCC CTT GTC ATC GTC GTC TTT ATA GTC CG	

Table S2 HPLC-Gradient used in this study.

Time [min]	Eluent A [%]	Eluent B [%]
1.50	70	30
8.00	17.5	82.5
11.50	0	100
13.50	0	100
14.00	70	30
18.00	70	30

Table S3 Chromatographic parameters for the quantification of the target compounds.

Compound name		Retention time [min]	λ_{max} [nm]	Calibration Parameters		
				a	b	r^2
1	Astaxanthin	6.5	480	141.002	9682.89	0.9999
2	Zeaxanthin	7.2	453	1397.65	4758.67	0.9973
3	Canthaxanthin	8.3	475	-1214.94	15655.5	0.9999
4	β-Carotene	11.5	450	-9041.02	399.705	0.9999

[a)] linear calibration, area [Au^2/ng], chromatogram traces: max-abs 400-500nm, fixed injection volume 5 µl.
